# Immunotherapy With Programmed Cell Death 1 Versus Programmed Cell Death Ligand 1 Inhibitors in Patients With Advanced Non–Small Cell Lung Cancers: A Multicenter, Retrospective Analysis

**DOI:** 10.1002/mco2.70476

**Published:** 2025-11-14

**Authors:** Jiadi Gan, Kaixin Lei, Tao Chang, Juan Wang, Ruiyuan Yang, Quanling Kong, Jingyi Yuan, Wenjun Meng, Yalun Li, Lina He, Jiaoming Li, Zeng Yan, Bojiang Chen, Weimin Li

**Affiliations:** ^1^ Department of Respiratory and Critical Care Medicine West China Hospital Sichuan University Chengdu Sichuan China; ^2^ Precision Medicine Research Center, Precision Medicine Key Laboratory of Sichuan Province, State Key Laboratory of Respiratory Health and Multimorbidity West China Hospital, Sichuan University Chengdu Sichuan China; ^3^ Institute of Respiratory Health Frontiers Science Center for Disease‐Related Molecular Network West China Hospital Sichuan University Chengdu Sichuan China; ^4^ West China School of Medicine West China Hospital Sichuan University Chengdu Sichuan China; ^5^ Department of Critical Care Medicine West China Hospital of Sichuan University Chengdu Sichuan China; ^6^ Department of Radiation Oncology The Affiliated Hospital of Qingdao University Qingdao Shandong China; ^7^ Department of Radiation Oncology Shandong Cancer Hospital and Institute Shandong First Medical University and Shandong Academy of Medical Sciences Jinan Shandong China; ^8^ Department of Pain Management West China Hospital Sichuan University Chengdu Sichuan China; ^9^ State Key Laboratory of Oncology in South People's Republic of China Collaborative Innovation Center for Cancer Medicine Sun Yat‐sen University Cancer Center Guangzhou Guangdong China; ^10^ Department of Neurosurgery Sichuan Clinical Research Center for Cancer Sichuan Cancer Center Sichuan Cancer Hospital & Institute University of Electronic Science and Technology of China Chengdu Sichuan China; ^11^ Department of Respiratory and Critical Care Medicine Chengdu First People's Hospital Chengdu Sichuan China

**Keywords:** immunotherapy, lung cancer, programmed cell death 1, programmed cell death ligand 1

## Abstract

This multicenter retrospective study analyzed data from 1266 patients with advanced non–small cell lung cancer (NSCLC) across five leading hospitals in China. The aim was to evaluate survival outcomes and safety profiles of programmed cell death 1 (PD‐1) and programmed cell death ligand 1 (PD‐L1) inhibitors. The main outcomes included overall survival (OS) and progression‐free survival (PFS), while the secondary endpoint was adverse events. Kaplan–Meier survival analysis, univariate and multivariate Cox regression modeling, and propensity score matching (PSM) analyses were performed to compare the real‐world efficacies of PD‐1 and PD‐L1. Patients receiving PD‐1 inhibitors had significantly longer median OS compared with those treated with PD‐L1 inhibitors (28.2 versus [vs.] 24.6 months; hazard ratio [HR] 0.74 [95% confidence interval (CI) 0.59–0.93]; *p* = 0.0099), with consistent effects after PSM analysis (HR 0.70 [95% CI 0.12–0.91]; *p* = 0.005) and multivariable, adjusted Cox regression model with HR of 0.74 ([95% CI 0.59–0.93]; *p* = 0.01). Further analysis indicated that body mass index ≥ 24 kg/m^2^ (HR 0.72 [95% CI 0.75–0.93]; *p* = 0.014) and history of hypertension (HR 1.35 [95% CI 1.01–1.79]; *p* = 0.037) may interfere with the therapeutic effects of PD‐1 with comparable safety profiles, which renewed personalized immunotherapy options for NSCLC patients in clinical settings.

## Graphical abstract

The graphic abstract presents a retrospective study focused on lung cancer immunotherapy, carried out across five hospitals. It evaluates the efficacy of PD‐1 versus PD‐L1 inhibitors in treating non–small cell lung cancer (NSCLC). The primary endpoints are overall survival (OS) and progression‐free survival (PFS), while the second endpoint is drug safety. The key findings reveal that PD‐1 inhibitors significantly OS compared to PD‐L1 inhibitors. Safety profiles were assessed without a significant difference. Characteristics including overweight and normal blood pressure are associated with better survival rates. Remarkably, PD‐1 inhibitors demonstrated superior survival outcomes, providing valuable insights into the strategic selection of immunotherapeutic agents for lung cancer treatment, ultimately aiming to improve patient prognosis and survival rates.

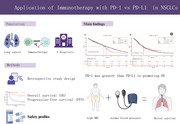

## 1 Introduction

Lung cancer is the leading cause of cancer‐related deaths worldwide, with NSCLC constituting approximately 85% of cases and driving substantial morbidity despite therapeutic advances [1, 2, 3]. The advent of immune checkpoint inhibitors targeting the PD‐1 and PD‐L1 axes has transformed the first‐line management of advanced NSCLC [4, 5, 6, 7, 8]; however, critical questions persist regarding the comparative efficacy of PD‐1 and PD‐L1 inhibitors. While landmark trials have established immunotherapy–chemotherapy combinations as standard care, divergent outcomes between agents have emerged. The KEYNOTE‐189 and KEYNOTE‐407 trials demonstrated the significant survival benefits of pembrolizumab (anti‐PD‐1) in non‐squamous and squamous NSCLC, respectively [7, 9], whereas IMpower131 and other trials failed to show improvement in overall survival (OS) with atezolizumab (anti‐PD‐L1) in squamous histology [7, 10, 11, 12]. Such discrepancies underscore the unresolved questions regarding the intrinsic differences between these therapeutic classes.

PD‐1 and PD‐L1 inhibitors disrupt the immune evasion axis and restore T cell‐mediated tumor clearance [13, 14, 15]. While both classes target the PD‐1/PD‐L1 pathway, emerging evidence suggests that the mechanistic differences are influenced by factors such as PD‐L1/PD‐L2 co‐expression [16], tumor glycosylation patterns [17], and pharmacokinetic properties [18]. Preclinical studies have indicated that PD‐1 inhibitors may elicit broader immune activation by blocking the interactions between PD‐L2 and CD80, whereas PD‐L1 inhibitors preserve PD‐L2 binding [19, 20, 21, 22]. However, the inherent mechanisms of PD‐1 and PD‐L1 may differ in their biological effects on antitumor therapies. PD‐1 inhibitors interrupt the binding of PD‐L1 and PD‐L2, whereas PD‐L1 inhibitors preserve PD‐1/PD‐L2 signaling, which has potential clinical implications given the prognostic role of PD‐L2 in NSCLC [16, 23]. Additional mechanisms, including CD80‐mediated co‐stimulation and differences in binding kinetics, may further distinguish antitumor activity.

In terms of the comparison between PD‐1 and PD‐L1 immunotherapy, the current evidence relies predominantly on cross‐trial comparisons and meta‐analyses of randomized controlled trials (RCTs), which suggest similar efficacy but lack the reliability to assess treatment effects across diverse patient subsets and combination regimens. Notably, most existing studies contrast PD‐1 and PD‐L1 inhibitors against placebo, chemotherapy, or targeted therapy, with relatively few directly comparing the two agents [24, 25, 26]. Although meta‐analyses of RCTs have performed indirect comparisons and found no significant differences in survival outcomes [22, 23, 24], these findings are constrained by the inherent limitations of explanatory RCTs in capturing real‐world heterogeneity. Therefore, well‐designed cohort studies are required to refine the understanding of these agents in a broader clinical context.

To fill this knowledge gap, this multicenter retrospective analysis, involved 1266 patients diagnosed with advanced NSCLC, treated with PD‐1 or PD‐L1 inhibitors across institutions in the Sichuan, Shandong, and Guangdong provinces of China. The aim was to assess the comparative effectiveness of PD‐1 and PD‐L1 blockades in real‐world practice by evaluating survival outcomes, safety, and subgroup‐specific responses to inform biomarker‐driven therapeutic selection. Comprehensive subgroup analyses were performed to identify potential beneficiary populations, and all adverse events (AEs) were systematically recorded to assess differences in treatment adherence. By leveraging real‐world data, this investigation sought to provide clinically relevant insights into the differential efficacy and safety of PD‐1 and PD‐L1 inhibitors and refine therapeutic strategies for the management of NSCLC.

## 2 Results

### 2.1 Baseline Characteristics

**FIGURE 1:**
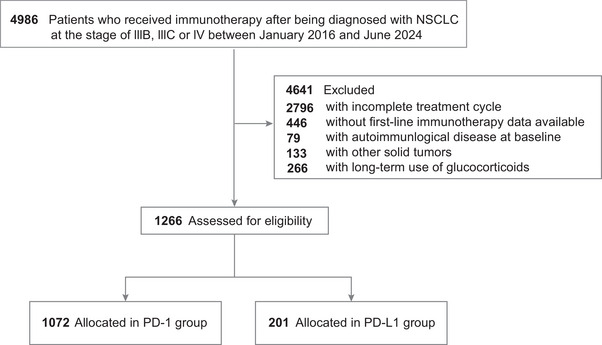
The flowchart of included samples and allocation.

Of an initial cohort of 4896 patients, 1266 were eligible for the final analysis, comprising 1079 individuals in the PD‐1 and 201 in the PD‐L1 group (Figure 1). The median age in the PD‐1 group was 61.8 years (range 42.8–80.7 years) and 62.5 years (range 43.8–81.1 years) in the PD‐L1 cohort. Among the PD‐1 cohort, 832 (78%) patients were male, 952 (89%) were diagnosed with Stage IV disease, 710 (66%) had a PD‐L1 tumor proportion score (TPS) < 1%, and 558 (52%) reported a smoking history (Table 1). Adenocarcinoma was prevalent in 706 (66%) patients, with 218 (20%) exhibiting *EGFR* mutations and 304 (28%) with *KRAS* mutations. Most patients had Eastern Cooperative Oncology Group (ECOG) scores between 0 and 2 (694 in the PD‐1 cohort and 104 in the PD‐L1 cohort, respectively). The treatment regimens included immunotherapy combined with chemotherapy in 712 patients (66%), triple therapy in 278 (26%), monotherapy in 61 (6%), and immunotherapy with antiangiogenic therapy in 21 (2%).

**TABLE 1:** Characteristics of included patients.

Characteristics	PD‐1 group (*n* = 1072)	PD‐L1 group (*n* = 201)	
	Patients, no. (%)	Patients, no. (%)	*p* value
Age, median (range)	61.8 (42.8–80.7)	62.5 (43.8–81.1)	0.359
Sex, no. (%)			0.112
Male	832 (78)	161 (80)	
Female	240 (22)	40 (20)	
BMI, median (range)	23.4 (16.7–30.1)	22.9 (15.9–29.8)	0.044
Smoking history			
Never	514 (48)	72 (36)	< 0.001
Ever	558 (52)	129 (64)	
Alcoholic history			0.436
Never	676 (63)	116 (58)	
Ever	396 (37)	85 (42)	
ECOG performance status			0.053
0	188 (17)	55 (27)	
1	694 (65)	104 (52)	
≥ 2	183 (17)	40 (20)	
Unknown	7 (1)	2 (1)	
Hypertension	212 (20)	25 (13)	0.037
Histology			< 0.001
Adenocarcinoma	706 (66)	81 (40)	
Squamous cell carcinoma	309 (29)	96 (48)	
Other	57 (8)	24 (12)	
*EGFR* mutation	218 (20)	35 (18)	0.524
*KRAS* mutation	304 (28)	65 (34)	0.175
Disease stages			< 0.001
IIIB, IIIC	120 (11)	56 (28)	
IVA, IVB	952 (89)	145 (72)	
PD‐L1 TPS %			0.772
< 1	206 (19)	45 (22)	
1–49	208 (20)	37 (18)	
> 50	138 (13)	20 (10)	
Unknown	520 (48)	99 (50)	
Metastasis			
Brain	543 (51)	129 (66)	< 0.001
Liver	422 (39)	119 (61)	< 0.001
Bone	575 (54)	130 (67)	< 0.001
Lung and pleura	703 (66)	158 (81)	< 0.001
Therapy line			0.978
First	325 (72.1)	103 (72.5)	
Second	94 (20.8)	27 (19.0)	
Third	32 (7.1)	12 (8.5)	
Therapy arrangement			0.034
IT	61 (6)	14 (7)	
IT + CT	712 (66)	145 (72)	
IT + AA	21 (2)	8(4)	
IT + CT + AA	278 (26)	34 (17)	

Abbreviations: AA, antiangiogenic agents; CT, chemotherapy; IT, immunotherapy; PD‐1, programmed cell death 1; PD‐L1, programmed cell death ligand 1; TPS, tumor proportion score.

In the PD‐L1 group, 161 (83%) patients were male, 145 (75%) were diagnosed with Stage IV disease, 138 (69%) had TPS < 1%, and 129 (66%) were smokers. Squamous cell carcinoma was identified in 96 (48%) patients, with *EGFR* mutations present in 35 (18%), and *KRAS* mutations in 65 (34%). Most of the 145 (72%) patients underwent combined immunotherapy and chemotherapy, 34 received additional antiangiogenic treatment, 14 received monotherapy, and 1 received immunotherapy with antiangiogenic agents. Invasive patterns affecting the lungs and pleura were common and noted in 703 (66%) and 158 (81%) patients in the PD‐1 treatment and PD‐L1 therapy, respectively.

### 2.2 Survival Outcomes in the PD‐1 and PD‐L1 Groups

**FIGURE 2:**
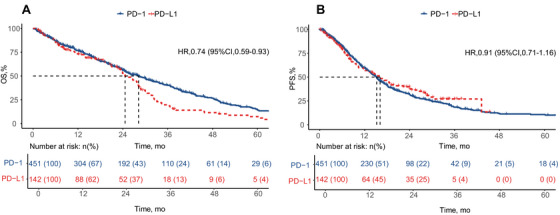
The estimated OS and PFS between PD‐1 group and PD‐L1 group. (A) The OS in PD‐1 and PD‐L1. (B) The PFS in PD‐1 and PD‐L1. OS, overall survival; PFS, progression‐free survival.

Following a median follow‐up of 19.9 months, there were no losses to follow‐up, and 593 patients were included in the survival analysis. Figure 2 shows that PD‐1 demonstrated a significantly greater effect on OS, irrespective of other variables, with a median OS of 28.2 months (95% CI 24.7–32.1; log‐rank *p* = 0.0099) (Figure 2A). In contrast, this significant effect was absent for PFS, for which the median time was 15.2 months (95% CI 13.6–16.6; log‐rank *p* = 0.49) (Figure 2B). For the PD‐L1 cohort, the median OS and PFS were 24.6 and 16.1 months, respectively.

### 2.3 PSM Analysis

**FIGURE 3:**
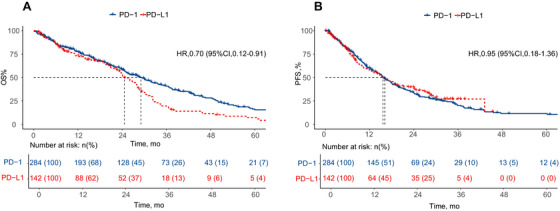
The survival outcomes of (A) OS and (B) PFS after PSM analysis. OS, overall survival; PFS, progression‐free survival; PSM, propensity score matching.

As shown in Table 2, after matching for age, sex, smoking history, hypertension history, and pathological classification with PD‐1:PD‐L1 = 2:1, 284 patients receiving PD‐1 therapy and 142 receiving PD‐L1 therapy were selected for the matching cohort, with baseline variables becoming comparable (Table S1). Kaplan–Meier univariate analysis revealed that patients treated with PD‐1 experienced significantly longer median OS than those treated with PD‐L1 therapy (29.1 versus [vs.] 24.6 months, *p* = 0.005) (Figure 3A). Meanwhile, intergroup PFS exhibited similarity (PD‐1 vs. PD‐L1, 15.6 vs. 16.1, respectively; *p* = 0.721) (Figure 3B), consistent with the pre‐propensity score matching (PSM) findings.

**TABLE 2:** Cox proportional regression models after adjustment for confounding factors.

PD‐1 vs. PD‐L1	HR	95% CI	*p* value
Model 1^a^	0.74	0.59–0.93	0.010
Model 2^b^	0.74	0.59–0.93	0.009
Model 3^c^	0.74	0.59–0.93	0.010

Abbreviations: CI, confidence interval; HR, hazard ratio; PD‐1, programmed cell death 1; PD‐L1, programmed cell death ligand 1.

^a^ Model 1 was the crude model without adjustment of any confounders.

^b^ Model 2 was adjusted for sex, age, body mass index, and smoking.

^c^ Model 3 accounted for sex, age, body mass index, smoking status, history of hypertension, pathological classification, and therapy lines.

### 2.4 Subgroup and Cox Regression Model Analyses

**FIGURE 4:**
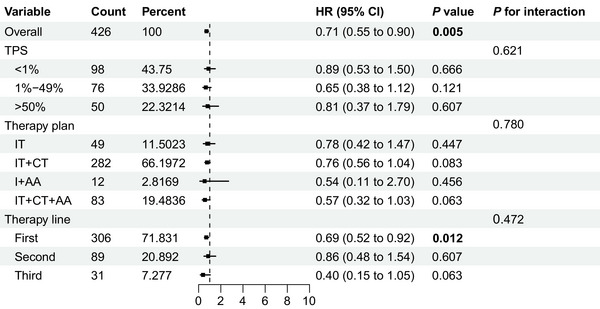
Subgroup analysis for OS based on TPS expression, therapy plans, and therapy lines. AA, antiangiogenic therapy; CT, chemotherapy; IT, immunotherapy; OS, overall survival; PFS, progression‐free survival; TPS, tumor proportion score.

**FIGURE 5:**
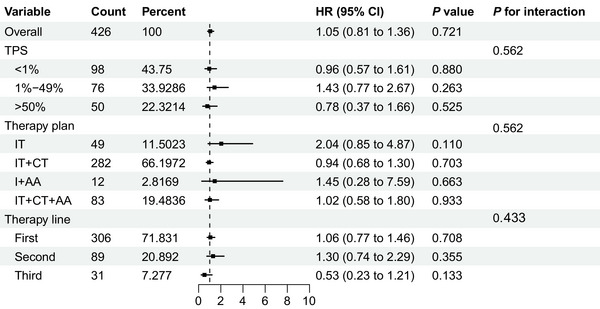
Subgroup analysis for PFS based on TPS expression, therapy plans, and therapy lines. AA, antiangiogenic therapy; CT, chemotherapy; IT, immunotherapy; OS, overall survival; PFS, progression‐free survival; TPS, tumor proportion score.

To validate the robustness of the findings, Cox proportional regression models were used to investigate the therapeutic differences between PD‐1 and PD‐L1 inhibitors (Table 2). In crude Model 1, PD‐1 significantly prolonged OS than PD‐L1 with HR of 0.74 ([95% CI 0.59–0.93]; *p* = 0.010). This finding remained consistent after adjustment for age, sex, BMI, and smoking in Model 2 and age, sex, BMI, smoking, history of hypertension, pathological classification, and therapy lines in Model 3, with HRs of 0.74 ([95% CI 0.59–0.93]; *p* = 0.010) for both models. Subgroup analysis was performed to examine the interactions between therapy lines, plans, and TPS in the matching cohort. As shown in Figures 4 and 5, subgroup analysis indicated that therapy plans, lines, and expression levels based on TPS did not influence the therapeutic effect of PD‐1 and PD‐L1 on both OS and PFS. In addition, the univariate and multivariate Cox stratification analyses identified potential prognostic factors for PD‐1 efficacy. Univariate Cox analysis was performed for all baseline variables (*p* < 0.1 as the selection criteria for multivariable Cox analysis). Ultimately, age, BMI, *EGFR* mutation status, histology, stage, sex, smoking status, hypertension, and metastasis were included in the multivariable analysis, which showed that overweight may be a beneficial factor for OS with HR of 0.72 ([95% CI 0.55–0.93]; *p* = 0.014) while hypertension may be a detrimental factor (HR 1.35 [95% CI 1.01–1.79]; *p* = 0.037) (Table S2, Figures S1 and S2).

### 2.5 Safety Analysis

Generally, pembrolizumab, sintilimab, and camrelizumab were the three most commonly used agents in the cohort (Table S3). PD‐L1 is seldom used compared with PD‐1, with most clinicians using durvalumab as an immunotherapeutic agent. Table 3 summarizes the AEs reported by the patients and recorded by the clinicians, demonstrating no significant differences between the treatment groups. During follow‐up, 475 toxic events were reported, of which 148 were deemed serious. Immune‐related AEs (irAEs) were noted in 30% patients receiving PD‐1 inhibitors and 31% of the PD‐L1 group, with severe cases occurring in 8% and 13% of patients, respectively. Pneumonia was the most common irAE, affecting 69 patients in the PD‐L1 group and 14 in the PD‐1 cohort, with severity observed in 18 PD‐1 and 6 PD‐L1 cases. Other prevalent irAEs, including dermatitis, allergies, and cardiac dysfunction, were more frequent in the PD‐1 group.

**TABLE 3:** Adverse events between PD‐1 group and PD‐L1 group.

	No. (%)				
	PD‐1 group		PD‐L1 group		*p* value
	Any grade	Grade ≥ 3	Any grade	Grade ≥ 3	0.44
irAEs					
All	124 (30)	33 (8)	24 (31)	10 (13)	
Dermatitis	13 (3)	2 (0)	4 (5)	3 (3)	
Pneumonia	69 (17)	18 (4)	14 (18)	6 (7)	
Cardiac dysfunction	11 (2)	1 (0)	2 (2)	1 (1)	
Adrenal insufficiency	4 (0)	0 (0)	1 (1)	0 (0)	
Kidney insufficiency	1 (0)	0 (0)	1 (1)	0 (0)	
Nervous system disorder	2 (0)	2 (0)	0 (0)	0 (0)	
Thyroid dysfunction	7 (1)	4 (0)	1 (1)	0 (0)	
Steroids use due to irAEs	6 (0)	3 (0)	0 (0)	0 (0)	
Endocrine disorder	1 (0)	1 (0)	0 (0)	0 (0)	
Colitis	1 (0)	0 (0)	1 (1)	0 (0)	
Allergic events	12 (2)	2 (0)	1 (1)	0 (0)	
Non‐irAEs					
All	278 (69)	90 (22)	49 (64)	15 (19)	
Myelosuppression	190 (47)	83 (20)	33 (43)	13 (17)	
Anemia	14 (3)	4 (0)	3 (3)	2 (2)	
Febrile	21 (5)	7 (1)	5 (6)	1 (1)	
Vomiting/nausea	24 (5)	3 (0)	6 (7)	0 (0)	
Diarrhea	7 (1)	0 (0)	2 (2)	1 (1)	
Cough	16 (3)	1 (0)	2 (2)	0 (0)	
Infectious events	5 (1)	2 (0)	4 (5)	2 (2)	
Liver dysfunction	14 (3)	3 (0)	5 (6)	0 (0)	
Digestive dysfunction	39 (9)	4 (0)	2 (2)	0 (0)	
Others	50 (12)	7 (1)	7 (9)	1 (1)	

Abbreviation: irAE, immune‐related adverse event.

Non‐irAEs were reported in 69% of patients receiving PD‐1 inhibitors and 64% of the PD‐L1 group, with myelosuppression being the most common, occurring in 47% of the PD‐1 group (83 severe cases) and 43% of the PD‐L1 group (13 severe cases). Digestive dysfunction occurred more frequently in the PD‐1 therapy than that in the PD‐L1 treatment.

## 3 Discussion

In this multicenter retrospective study involving 1266 patients with advanced NSCLC, PD‐1 inhibitors demonstrated a statistically significant survival benefit over PD‐L1 inhibitors, with a median OS of 28.2 months compared to 24.6 months (HR 0.74 [95% CI 0.59–0.93]), respectively, reinforcing PD‐1 blockade as the preferred initial therapeutic strategy. Despite the higher proportion of patients with Stage IV disease in the PD‐1 cohort (89% vs. 72% in the PD‐L1 cohort), which may reflect real‐world clinical preferences for PD‐1 inhibitors in advanced disease management [27, 28], our findings remained robust after PSM and multivariate adjustments, confirming the therapeutic superiority of PD‐1 inhibitors.

This survival disparity likely originates from mechanistic differences between inhibitors targeting PD‐1 and those targeting PD‐L1. While PD‐L1 inhibitors selectively block PD‐1/PD‐L1 interactions, PD‐1 inhibitors disrupt both the PD‐L1 and PD‐L2 pathways, an important distinction given that 23% of our cohort exhibited PD‐L2 expression, a biomarker associated with resistance to PD‐L1‐targeted therapies [15, 29]. Preclinical studies suggest that PD‐L2 engagement sustains immunosuppressive signaling through alternative receptors, such as RGMb, which may attenuate PD‐L1 inhibitor efficacy [30, 31]. Conversely, PD‐L1 inhibitors uniquely inhibit the PD‐L1/CD80 axis, potentially enhancing dendritic cell‐mediated antigen presentation in treatment‐naïve microenvironments [22, 32].

Subgroup analyses revealed that patients without hypertension and those who are overweight may benefit more profoundly from PD‐1 inhibitors. Hypertension appears to be a detrimental factor in patients receiving PD‐1 therapy, which may be related to the significant number of targeted therapies in our cohort. Hypertension is a well‐recognized adverse effect of targeted treatments that can exacerbate preexisting hypertension or cause new‐onset hypertension. These conditions often lead to vascular endothelial dysfunction, consequently affecting the immunomodulatory efficacy of PD‐1 inhibitors [33, 34]. In addition, a recent meta‐analysis found that treatment with PD‐1 inhibitors is associated with an increased incidence of cardiovascular toxic events, including hypertension. Although cardiovascular AEs were rare in our study, they provide a possible explanation for the significantly reduced benefits observed in patients with hypertension [35]. Interestingly, although overweight status is a risk factor for NSCLC, it appears to enhance the responsiveness of patients with advanced NSCLC to immunotherapy. Studies have shown that in patients with obesity, adipocytes secrete estrogen, leptin, and inflammatory factors that regulate the expression of PD‐1 on the surface of macrophages and CD8‐positive T cells, thereby promoting responsiveness to immunotherapy [36, 37, 38]. Paradoxically, an *EGFR*/*KRAS* mutation status did not predict differential outcomes, a finding discordant with those reported in previous studies [20, 39], which may reflect confounding by concurrent STK11/LKB1 alterations or limited subgroup sample sizes (*n* = 523 tested).

After PSM, no significant differences were found in treatment line, treatment regimen, or TPS expression levels, suggesting that the superior efficacy of PD‐1 inhibitors on OS was not influenced by these variables. Results of this study, based on a multicenter, real‐world population, imply that PD‐1 may be the preferred treatment option for achieving superior survival benefits, irrespective of treatment line, regimen, or TPS. In the univariate analysis for OS, we observed that the survival curves initially overlapped during treatment and diverged over time. The impactful KEYNOTE‐189 study observed an evident therapeutic effect of PD‐1 inhibitor, pembrolizumab, compared with traditional chemotherapy, which seemed to conflict with the present findings [7]. However, the study population is heterogeneous. The KEYNOTE‐189 study strictly included patients diagnosed with non‐squamous NSCLC and specifically excluded individuals with *EGFR* and *KRAS* mutations. In contrast, our study included a significant portion of patients with squamous cell carcinoma and those with *EGFR* and *KRAS* mutations, which are likely contributing to this difference and warrant further clinical and translational research for confirmation. In addition, Lu et al. [40] assessed how the timing of immunotherapy initiation affects the prognosis of patients with NSCLC and found that a delayed combination of PD‐1 with chemotherapy could result in greater survival benefits. Thus, we hypothesized that PD‐1/PD‐L1 treatment may exhibit a delayed response effect, which could explain the later divergence of the survival curves [41, 42]. Safety profiles were similar across the groups, with irAEs occurring in 30% of PD‐1 and 31% of PD‐L1 inhibitor recipients. Myelosuppression (47% vs. 43%) and pneumonia (17% vs. 18%) were critical toxicities requiring proactive monitoring [24, 39]. The absence of hypertension, a condition linked to chronic endothelial inflammation and T‐cell dysfunction, was correlated with improved PD‐1 inhibitor efficacy, suggesting that baseline vascular health may modulate immunotherapy outcomes [39].

The present study represents the first large‐scale, real‐world, comparative analysis of PD‐1 and PD‐L1 inhibitors in advanced NSCLC, revealing novel survival hierarchies and clinically actionable distinctions. We identified PD‐1 inhibitors as the preferred first‐line strategy, particularly for patients with brain metastases and smokers, mechanistically linked to dual PD‐L1/PD‐L2 blockade, a paradigm that advances the biological rationale for differential efficacy. In addition, we revealed the transient superiority of PD‐L1/antiangiogenic combinations in the early lines, highlighting time‐dependent microenvironmental interactions. By integrating comprehensive subgroup analyses and safety validations without incremental toxicity risks, we established biomarkers for precision sequencing and challenged the interchangeability assumption of PD‐1/PD‐L1 inhibitors, offering transformative insights for guideline refinement and personalized therapeutic decision‐making.

Despite the insights from this large, real‐world cohort, the limitations of this study warrant consideration. The retrospective design potentially introduced selection bias, which was exacerbated by greater representation in the PD‐1 group (i.e., 85% of the cohort). While our findings align with trends in randomized trials, prospective studies are needed to validate the subgroup‐specific benefits and clarify the underlying mechanisms, particularly the role of CD80 modulation in PD‐L1 monotherapy efficacy.

In conclusion, this study established PD‐1 inhibitors as an optimal first‐line therapy for advanced NSCLC, particularly among smokers and patients with brain metastases. The temporal efficacy of PD‐L1/antiangiogenic combinations and the unique activity of PD‐L1 monotherapy highlight the need for biomarker‐driven strategies. Future trials that integrate PD‐L2 and CD80 assessments may further refine patient selection and therapeutic sequencing.

## 4 Methods

### 4.1 Patient Selection

This retrospective study included patients diagnosed with advanced NSCLCs, who underwent PD‐1 or PD‐L1 inhibitors treatment between January 2016 and June 2024 at 5 centers in China: 3 in Sichuan (West China Hospital of Sichuan University [*n* = 366]; Sichuan Cancer Hospital [*n* = 87]; Chengdu First People's Hospital [*n* = 74]); 1 in Shandong (Shandong Cancer Hospital [*n* = 680]); and 1 in Guangzhou (Sun Yat‐sen University Cancer Center [*n* = 66]). Survival outcome data were unavailable for patients from Shandong Cancer Hospital; as such, these cases were allocated solely in the safety analysis.

Eligible patients were required to have histopathologically confirmed advanced or metastatic NSCLC, and radiological evidence of metastasis assessed using computed tomography (CT) and/or magnetic resonance imaging (MRI). In addition, patients must have undergone ≥ 1 cycle(s) of immunotherapy with PD‐1 or PD‐L1 inhibitors. Patients diagnosed with severe autoimmune disorders, required long‐term glucocorticoid therapy, or had other advanced solid tumors were excluded.

All patients enrolled underwent anti‐PD‐1 or anti‐PD‐L1 therapy, either as monotherapy or in combination with chemotherapy, antiangiogenic therapy, or a combination of both, irrespective of the treatment line. PD‐1/PD‐L1 inhibitors and other antineoplastic agents were administered at recommended dosages according to drug labels or clinical trial protocols. Ethical approval for the study protocol was obtained from the ethics committees of all participating centers.

### 4.2 Data Collection

Patient information was retrieved from the electronic health records of the respective medical centers. The collection of follow‐up data adhered to uniform criteria, including responses to different treatments and clinical outcomes. Baseline characteristics included age, sex, smoking history, alcohol consumption history, histological classification, driver gene mutation status, World Health Organization (WHO) classification, PD‐L1 expression, and metastasis status. The clinical characteristics of age, sex, smoking, alcohol consumption history, WHO classification, and metastasis status were documented at the initial diagnosis, whereas histological classification, driver gene mutation status, and PD‐L1 level were obtained from pathology reports.

The primary outcomes of this study were OS and PFS. OS was defined as the interval between diagnosis and death from any cause, whereas PFS was defined as the interval from treatment initiation to histological or radiological disease progression or the occurrence of new metastases. Patients who were alive or experience disease progression at the last follow‐up visit were censored at the last recorded clinical visit.

AEs were documented by physicians or through a self‐reporting system. In cases of patient noncompliance with medication, treatment modifications were made upon approval from the attending oncologist(s). AEs were classified according to the National Cancer Institute's Common Terminology Criteria for Adverse Events (CTCAE), version 5.0. Both irAEs and non‐irAEs of all grades were recorded, with those of grade ≥ 3 classified as serious AEs.

This study received approval from the Institutional Review Board of the West China Hospital of Sichuan University (approval number 20241410), and written informed consent was obtained from all patients before participation. This multicenter observational study was conducted in compliance with the World Medical Association's Declaration of Helsinki [43] and adhered to the reporting guidelines for case series [44].

### 4.3 Statistical Analysis

#### 4.3.1 Data Presentation and Kaplan–Meier Analysis

Continuous data are expressed as median (interquartile range), whereas categorical variables are shown as the percentage of the corresponding category. For intergroup comparisons, the Wilcoxon rank‐sum test was used for categorical data, whereas the chi‐squared (*χ* ^2^) test was used for categorical variables. Kaplan–Meier methods were used to estimate OS and PFS, with statistical significance determined using log‐rank tests.

#### 4.3.2 Sensitivity and Subgroup Analyses

To mitigate the potential selection bias inherent in retrospective study designs, multiple statistical methods were used to validate the findings of Kaplan–Meier analysis. First, PSM, age, sex, BMI, smoking history, hypertension, pathological type, and line of therapy were used as matching factors. A matching ratio of PD‐1:PD‐L1 (2:1) was used to ensure comparability between the groups. After PSM, baseline data analysis and Kaplan–Meier univariate analysis were conducted to verify the reliability of the findings. In addition, multivariable Cox analysis based on 3 models adjusted for different covariates was performed to further investigate the effects of PD‐1 and PD‐L1. To further observe possible prognostic factors, univariate and multivariable Cox analyses were performed. All statistical analyses were conducted by using R software (version 4.2.2, R Core Team; R Foundation for Statistical Computing, Vienna, Austria) and SAS, version 9.4 (SAS Institute, Cary, NC, USA). All statistical tests were two‐sided, and differences with *p* value less than 0.05 were considered to be significant.

## Author Contributions

Bojiang Chen, Weimin Li, and Jiadi Gan had full access to all data in the study and take responsibility for the integrity of the data and the accuracy of the data analysis. Concept and design: Bojiang Chen, Weimin Li, and Jiadi Gan. Data acquisition: Weimin Li, Juan Wang, Wenjun Meng, Yalun Li, Lina He, Jiaoming Li, and Zeng Yan. Data analysis: Jingyi Yuan, Ruiyuan Yang, and Tao Chang. Drafting of the manuscript: Kaixin Lei and Jiadi Gan. Critical review of the manuscript for important intellectual content: Quanling Kong, Kaixin Lei, Jingyi Yuan, and Juan Wang. Statistical analysis: Quanling Kong and Kaixin Lei. Administrative, technical, or material support: Bojiang Chen, Weimin Li, and Jiadi Gan. Supervision: Bojiang Chen and Weimin Li. All the authors have read and approved the final version of the manuscript submitted for publication.

## Funding

Fundamental Research Funds for the Central Universities (grant number: SCU2024D017); State Key Laboratory of Respiratory Health and Multimorbidity, State Key Laboratory (grant number: 2060204); 1•3•5 Project of State Key Laboratory of Respiratory Health and Multimorbidity, West China Hospital, Sichuan University (grant number: RHM2404); and 1•3•5 Project for Disciplines of Excellence, West China Hospital, Sichuan University (grant number ZYGD22009) provided financial support to Weimin Li. Sichuan Natural Science Foundation of Science Fund (2024NSFJQ0051) provided funding for Bojiang Chen.

## Ethics Statement

Clinical data were approved by the West China Hospital of Sichuan University. The protocol was approved by the leading ethics committee of the West China Hospital of Sichuan University (approval number [2024]1410). The study was conducted in compliance with the principles of the Declaration of Helsinki, and all procedures were performed in compliance with the relevant guidelines and regulations.

## Conflicts of Interest

The authors declare no conflicts of interest.

## Supporting information



Table S1 Baseline characteristics after PSM.
**Supporting Table S2**: Univariate and multivariate analysis in OS after PSM.
**Supporting Table S3**: Detailed information on drug use in PD‐1 and PD‐L1 cohort analysis.
**Supporting Figure S1**: Survival curves for OS in overweight patients (BMI ≥ 25).
**Supporting Figure S2**: Survival curves for OS in hypertensive patients.

## Data Availability

Data from this study, including de‐identified participant data, will be made available to others upon reasonable request. Additional documents, including the study protocol, statistical analysis plan, and informed consent form, are also available. Data are available from the corresponding author (weimin003@163.com, cjhcbj@outlook.com) upon reasonable request.
