# Supplementary material for: Immunotherapy With Programmed Cell Death 1 Versus Programmed Cell Death Ligand 1 Inhibitors in Patients With Advanced Non–Small Cell Lung Cancers: A Multicenter, Retrospective Analysis
Source: MedComm (2020). 2025 Nov 14;6(12):e70476. doi: 10.1002/mco2.70476 (PMC12618853; doi:10.1002/mco2.70476)
Supplement: Supplementary file 1 — Table S1 Baseline characteristics after PSM. Supporting Table S2: Univariate and multivariate analysis in OS after PSM. Supporting Table S3: Detailed information on drug use in PD‐1 and PD‐L1 cohort analysis. Supporting Figure S1: Survival curves for OS in overweight patients (BMI ≥ 25). Supporting Figure S2: Survival curves for OS in hypertensive patients. [file MCO2-6-e70476-s001.docx]

Supplement Materials

Table S1 Baseline characteristics after PSM

| **Characteristics** | **PD-1 group**  **(n= 284)** | **PD-L1 group**  **(n= 142)** | ***P* value** |
| --- | --- | --- | --- |
|  | Patients, No. (%) | Patients, No. (%) |  |
| **Age, median (range)** | 61.7 (23.0, 84.0) | 64.0 (31.0, 83.0) | 0.844 |
| **Sex,n (%)** |  |  | 0.997 |
| Male | 221 (77.8) | 111 (78.2) |  |
| Female | 63 (22.2) | 31 (21.8) |  |
| **BMI, median (range)** | 22.7 (14.7-31.8) | 22.6 (15.6-32.3) | 0.867 |
| **Smoking history** |  |  | 0.998 |
| Never | 127 (44.7) | 64 (45.1) |  |
| Ever | 157 (55.3) | 78 (54.9) |  |
| **Alcoholic history** |  |  | 0.435 |
| Never | 190 (66.9) | 86 (60.6) |  |
| Ever | 94 (33.1) | 56 (39.4) |  |
| **Hypertension** | 73 (25.7) | 34 (23.9) | 0.925 |
| **ECOG performance status** |  |  | 0.946 |
| 0 | 90 (31.7) | 52 (36.6) |  |
| 1 | 144 (50.7) | 67 (47.2) |  |
| ≥2 | 45 (15.8) | 22 (15.5) |  |
| **Unknown** | 5 (1.8) | 1 (0.7) |  |
| **Histology** |  |  | 0.977 |
| Adenocarcinoma | 137 (48.2) | 66 (46.5) |  |
| Squamous cell carcinoma | 119 (41.9) | 57 (40.1) |  |
| Other | 28 (9.9) | 19 (13.4) |  |
| **Disease stages** |  |  | 0.248 |
| IIIB, IIIC | 77 (27.1) | 28 (19.7) |  |
| IVA, IVB | 207 (72.9) | 114 (80.3) |  |
| **EGFR+** | 36 (12.7) | 16 (11.3) | 0.598 |
| **KRAS+** | 19 (6.7) | 8 (5.6) | 0.904 |
| **PD-L1 TPS %** |  |  | 0.916 |
| <1 | 64 (22.5) | 34 (23.9) |  |
| 1~49 | 35 (12.3) | 15 (10.6) |  |
| >50 | 46 (16.2) | 30 (21.1) |  |
| Unknown | 139 (48.9) | 63 (44.4) |  |
| **Metastasis** |  |  |  |
| Brain | 33 (11.6) | 22 (15.5) | 0.532 |
| Liver | 24 (8.5) | 13 (9.2) | 0.971 |
| Bone | 67 (23.6) | 31 (21.8) | 0.921 |
| Lung and pleura | 161 (56.7) | 158 (62.7) | 0.497 |
| **Therapy line** |  |  | 0.941 |
| **First** | 203 (71.5) | 103 (72.5) |  |
| **Second** | 62 (21.8) | 27 (19.0) |  |
| **Third** | 19 (6.7) | 12 (8.5) |  |
| **Therapy arrangement** |  |  | 0.918 |
| IT | 29 (10.2) | 20 (14.1) |  |
| IT+CT | 193 (68.0) | 89 (62.7) |  |
| IT+AA | 7 (2.5) | 5 (3.5) |  |
| IT+CT+AA | 55 (19.4) | 28 (19.7) |  |

Abbreviations: PD-1, programmed cell death 1; PD-L1, programmed death-ligand 1; TPS, tumor proportion score; IT, immunotherapy; CT, chemotherapy; AA, anti-angiogeni agents

Table S2 Univariate and multivariate analysis in OS after PSM.

|  | **OS** | | | |
| --- | --- | --- | --- | --- |
|  | **Univariate** | | **Multivariate** | |
|  | HR (95%CI) | *P* value | HR (95%CI) | *P* value |
| Age<65 years | Reference |  | Reference |  |
| Age≥65 years | 1.29 (1.02-1.62) | 0.032 | 1.07 (0.82-1.39) | 0.634 |
| BMI<25 kg/m^2^ | Reference |  | Reference |  |
| BMI≥25kg/m^2^ | 0.75 (0.58-0.97) | 0.026 | 0.72 (0.55-0.93) | 0.014 |
| ECOG=0 | Reference |  | Reference |  |
| ECOG=1 | 1.22 (0.97-1.54) | 0.093 | 1.26 (0.99-1.59) | 0.052 |
| With metastasis | Reference |  | Reference |  |
| With no metastasis | 1.32 (0.97-1.81) | 0.077 | 1.21 (0.88-1.66) | 0.246 |
| With no hypertension | Reference |  | Reference |  |
| history of hypertension | 1.37 (1.05-1.79) | 0.019 | 1.35 (1.01-1.79) | 0.037 |
| Without radiotherapy | Reference |  | Reference |  |
| Radiotherapy | 1.44 (1.08-1.92) | 0.012 | 1.28 (0.92-1.79) | 0.141 |
| Second- and third-line immunotherapy | Reference |  | Reference |  |
| First-line immunotherapy | 1.32 (1.01-1.73) | 0.045 | 1.13 (0.83-1.54) | 0.438 |
| First- and second-line therapy | Reference |  | Reference |  |
| Third-line immunotherapy | 0.64 (0.40-1.04) | 0.069 | 0.71 (0.4101.23) | 0.224 |

Table S3 Detailed information on drug use in PD-1 and PD-L1 cohort analysis

| Drug name | Category | Therapeutic line | Number |
| --- | --- | --- | --- |
| Sintilimab | PD-1 | 1 | 83 |
| Sintilimab | PD-1 | 2 | 25 |
| Sintilimab | PD-1 | 3 | 8 |
| Tisleizumab | PD-1 | 1 | 3 |
| Tisleizumab | PD-1 | 2 | 23 |
| Tisleizumab | PD-1 | 3 | 5 |
| Toripalimab | PD-1 | 1 | 11 |
| Toripalimab | PD-1 | 2 | 5 |
| Toripalimab | PD-1 | 3 | 1 |
| Slulimumab | PD-1 | 1 | 1 |
| Sugemalimab | PD-L1 | 1 | 1 |
| Pembrolizumab | PD-1 | 1 | 120 |
| Pembrolizumab | PD-1 | 2 | 22 |
| Pembrolizumab | PD-1 | 3 | 5 |
| Nivolumab | PD-1 | 1 | 8 |
| Nivolumab | PD-1 | 2 | 14 |
| Nivolumab | PD-1 | 3 | 5 |
| Camrelizumab | PD-1 | 1 | 98 |
| Camrelizumab | PD-1 | 2 | 12 |
| Camrelizumab | PD-1 | 3 | 2 |
| Durvalumab | PD-L1 | 1 | 43 |
| Durvalumab | PD-L1 | 2 | 4 |
| Durvalumab | PD-L1 | 3 | 2 |
| Atezolizumab | PD-L1 | 1 | 84 |
| Atezolizumab | PD-L1 | 2 | 6 |
| Atezolizumab | PD-L1 | 3 | 1 |
| Adebrelimab | PD-L1 | 1 | 1 |

Supplement Figures


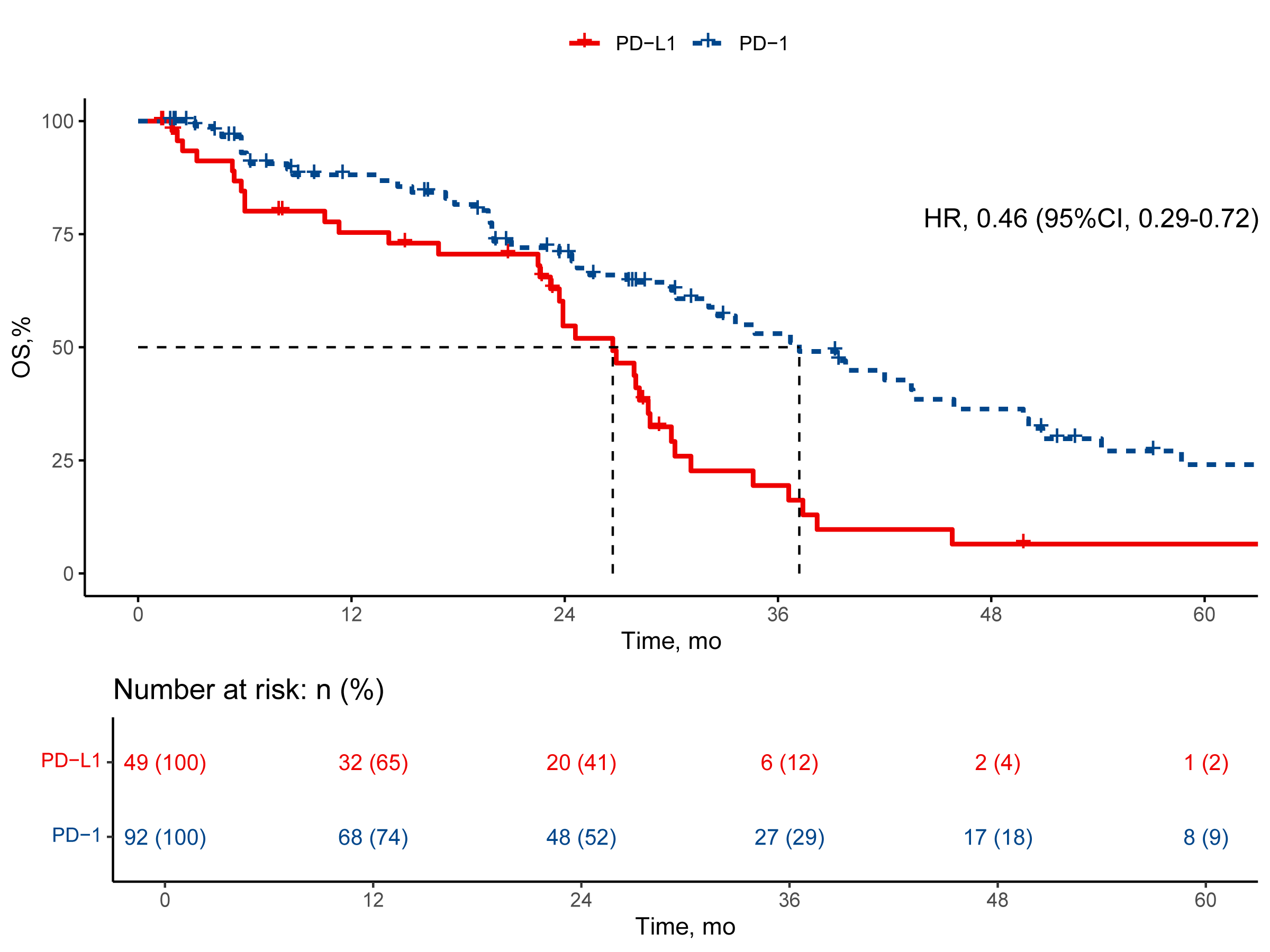


Figure S1 Survival curves for OS in overweight patients (BMI ≥ 25)


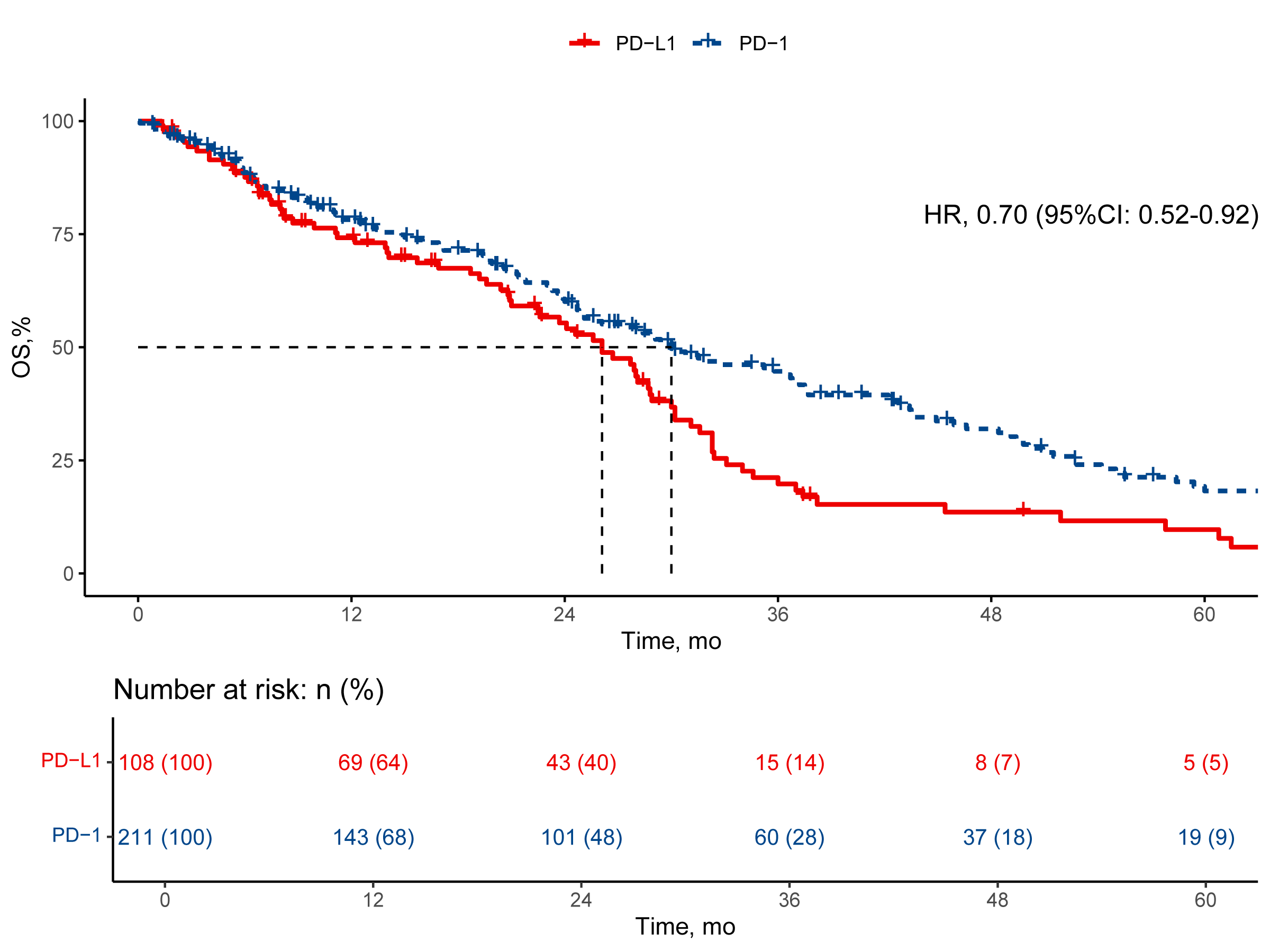


Figure S2 Survival curves for OS in hypertensive patients.
